# School-Based Interventions and Programs to Address Weight Issues

**DOI:** 10.1155/2018/3538964

**Published:** 2018-10-28

**Authors:** Moonseong Heo, Angelo Pietrobelli, Judith Wylie-Rosett, Myles S. Faith

**Affiliations:** ^1^Department of Public Health Sciences, Clemson University, Clemson, SC, USA; ^2^Department of Pediatrics, University of Verona, Verona, Italy; ^3^Pennington Biomedical Research Center, Baton Rouge, LA, USA; ^4^Department of Epidemiology and Population Health, Albert Einstein College of Medicine, Bronx, NY, USA; ^5^Department of Counseling, School, and Educational Psychology, Graduate School of Education, University at Buffalo-SUNY, Buffalo, NY, USA

School life is essential for children and adolescents for education and beyond; it also is their “job” and schools are the places where they spend a majority of their time. Moreover, parents care greatly about their children's school environment (e.g., safety and teacher quality), academic performance, and general well-being status, monitoring all of these components carefully and critically. Thus, schools are ideally suited to serve as a real-world experimental laboratory for interventions targeting on pediatric weight-related issues. The external validity and, arguably, number of stakeholders involved could not be higher in striving to curb alarming obesity prevalence globally.

School-based educational and participatory approaches, in conjunction with school or community changes to promote healthy nutrition and physical activities, can effectively change student behaviors and in some studies weight status outcome. Such approaches might ultimately help students enhance academic achievements as there is an inverse association between children's weight status and academic achievement. To this end, spheres of influence that need to be considered in implementing school obesity assessment or intervention include attitudes and social/cultural norms about obesity and health at the individual student/family level, at the class/school/district level, and at the broader levels of community identity.

It is, therefore, natural that school-based trials often call for multidisciplinary collaborations to take on the problem involving multiple stakeholders such as students themselves, parents, teachers, and intra- or extramural committees and organizations. This dimensionality presents great opportunities and challenges at the same time. It is challenging to tailor school-based obesity prevention program by addressing personal and environmental factors, in addition to basic knowledge and skills. It is even more so since schools exemplify dynamic social systems that are living and “growing” rather than do merely a physical, concrete, or static entity. As such, applying principles of participatory research can help develop partnerships of trust, shared vision, and mutual capacity building. Such partnerships would yield genuine community engagement at multiple levels in order for students to achieve healthier lifestyles and body weights.

We believe that the papers in this special issue challenged the field of obesity in the contexts addressed above. D. W. Pittman et al.'s “Boss' Healthy Buddies” study implemented a nutrition education program engaging students, teachers, and families as a part of a South Carolina elementary school health education curriculum. The program was more effective than adopting a commercially available similar program at a school level. K. M. Morrison et al. demonstrated that physical activity participation among Canadian elementary school students are influenced by “perceived athletic competence,” which depends in part on evaluation of oneself relative to peers. K. E. Finn et al. suggested that greater classroom engagements among high school students with higher BMI might help accomplish their academic potential and called for interventions to that end.

The papers herein also illustrated how school systems and their connection to obesity are part of a larger nested network. This network extends downward to biology and outwards to the community. For instance, G. A. do Nascimento et al. showed that physical exercise did not influence the association between FTO genotype and insulin metabolic outcomes among Brazilian overweight and obese students participating in intensive after-school exercise programs. On the other hand, J. Rieder et al. focused on effective implementation strategies of a middle-school-based comprehensive wellness program with realistic and timely goals. This program was implemented in an urban inner-city community-based setting by facilitating communications and developing trust among stakeholders, students, and families. Lastly, appropriate design and statistical analysis are critical to obtain valid and reliable knowledge from any sort of trials including school-based trials. In their review, M. Heo et al. noted that the inherent clustering or design effects need to be more fittingly accounted for when designing and analyzing school-based trial data.

In sum, this special issue reflects and confirms that school-based trials for addressing weight-related issues are an ever evolving and challenging research field that strongly calls for participation of multiple layers of stakeholders beyond students to maximize prevention and intervention effects. It remains an open question as to how the ubiquitously available online social networking platforms and applications could be reliably utilized to further facilitate school-based trials and collect data germane to the weight-related concerns.

